# Stress-symptoms and well-being in children and adolescents: factor structure, measurement invariance, and validity of English, French, German, Russian, Spanish, and Ukrainian language versions of the SSKJ scales

**DOI:** 10.1080/21642850.2021.1990062

**Published:** 2021-10-14

**Authors:** Vera Gillé, Denise Kerkhoff, Uwe Heim-Dreger, Carl-Walter Kohlmann, Arnold Lohaus, Heike Eschenbeck

**Affiliations:** a Department of Psychology, University of Education Schwäbisch Gmünd, Schwäbisch Gmünd, Germany; b Department of Psychology, University of Konstanz, Konstanz, Germany; c Faculty of Psychology and Sports Science, Bielefeld University, Bielefeld, Germany

**Keywords:** Stress symptoms, children, adolescents, cross-cultural, gender, measurement invariance

## Abstract

**Objective:**

The present cross-cultural study examined the factor structure, measurement invariance, and convergent validity of the Stress-Symptom and Well-Being Scales from the Stress and Coping Questionnaire for Children and Adolescents (SSKJ), originally in German, across gender and for five newly developed language versions: English, French, Russian, Spanish, and Ukrainian.

**Design:**

Children and adolescents (*N* = 5,227) from Germany, France, Russia, the Dominican Republic, Ukraine, and several English-speaking countries participated in the survey study.

**Main outcome measures:**

The SSKJ Stress-Symptom and Well-Being Scales capture stress symptomatology and well-being with five subscales: Somatic Symptoms, Anger, Sadness, Anxiety, and Well-Being. The Strengths and Difficulties Questionnaire (SDQ) was used for validation.

**Results:**

The factorial structure (five factors) was confirmed. In multi-group comparisons, confirmatory factor analyses showed partial metric invariance across the different languages. Regarding gender, results showed scalar invariance for all languages, except for Spanish. Gender differences were shown with girls scoring higher on somatic symptoms, sadness, anxiety (German-, French-, Russian-speaking samples), anger (French), and well-being (German, Ukrainian). Correlations with indicators of mental health and behavioral problems demonstrated convergent validity.

**Conclusion:**

The SSKJ Stress-Symptom and Well-Being Scales showed psychometric evidence for equivalence across the different languages and gender. Thus, this instrument is a useful tool for cross-cultural research in children and adolescents.

Stress is part of everyday life among children and adolescents (Kraag, Zeegers, Kok, Hosman, & Abu-Saad, 2006). Experiencing increased demands without the availability of adequate resources or coping strategies can be associated with the occurrence of stress-related symptoms (Lazarus & Folkman, 1984). This study deals with the subjective stress experience of children and adolescents from various countries and the stress-symptoms they report. For example, following the cross-national study, *Health Behaviour in School-aged Children* (HBSC), which surveyed 15-year-old adolescents in 37 countries, on average about 4 in 10 adolescents reported psychosomatic complaints such as headaches or trouble sleeping, indicating stress (including England with 51% reporting two or more stress-symptoms, France 49%, Wales 46%, Ukraine 45%, Ireland 42%, Russia 34% or Germany 33%; Walsh et al., 2020). However, for more detailed analyses of stress-symptoms, a more differentiated screening instrument for children and adolescents, available in various languages, is essential.

With regard to symptom measurement among children and adolescents, there are numerous assessments, most of which focus, from a more clinical perspective, on the occurrence of symptoms pointing to childhood psychopathology (for reviews on pediatric measures of psychosocial adjustment and mental health, see Deighton et al., 2014; Holmbeck et al., 2008). Widespread screening instruments for behavioral and emotional problems, for example, are the Conners’ Rating Scales (CRS-R; Conners, 2001), the Strengths and Difficulties Questionnaire (SDQ; Goodman, 1997), or the Youth Self-Report (YSR 11-18; Achenbach & Rescorla, 2001). These instruments are used internationally and are available in numerous language versions.

With significantly less reference to psychopathology and, moreover, often referred to as ‘stress-related’ health complaints (e.g. Eriksson & Sellström, 2010; Torsheim & Wold, 2001), the HBSC study measures with a brief 8-item symptom checklist (refering to the last 6 months, available in several language versions predominantly used in Europe and North America) somatic complaints (headache, stomachache, backache, dizziness) and psychological complaints (feeling low/depressed, irritable/bad-tempered, nervous, and having sleep problems; Gariepy, McKinnon, Sentenac, & Elgar, 2016). However, for the HBSC symptom checklist, a two-factor solution in which items would be more differentially divided into somatic and psychological subscales was not recommended (Ravens-Sieberer et al., 2008). Looking at psychological symptoms in a more differentiated way, anger, sadness, and anxiety are among the central facets of negative psychological experiences. For example, Watson and Tellegen (1985) postulated two independently varying dimensions of emotional state: positive and negative affect. Negative affect is characterized by nervousness, fearfulness or hostility, and low positive affect by lethargy and sadness. To measure both positive and negative affect, the Positive and Negative Affect Schedule (PANAS; Watson, Clark, & Tellegen, 1988) a self-report scale, was developed. The instrument is widely used as it has been translated into several languages and was also adapted for use with children and adolescents (e.g. Laurent et al., 1999; Wróbel, Finogenow, Szymańska, & Laurent, 2019). Overall, numerous childhood symptom scales are available. However, these are often more psychopathology-oriented scales for the assessment of psychosocial adjustment and mental health; checklists and scales for the recording of symptoms with less reference to clinical diagnoses show, for example, little differentiation between somatic and psychological subscales as well as within psychological symptoms (e.g. HBSC checklist), or they do not include scales for somatic symptoms (e.g. PANAS). For effective health promotion and early intervention in children and adolescents (e.g. screening, program evaluation), theory-driven, easily understandable scales whose item contents are oriented to everyday experience (in contrast to mental disorders and psychopathology) and which allow differentiation of psychologcial and somatic symptoms would be valuable.

Scales genuinely developed within the context of stress and coping, which additionally differentiate between psychological and somatic symptoms are the Stress-Symptom Scales from the Stress and Coping Questionnaire for Children and Adolescents (SSKJ 3-8; Lohaus, Eschenbeck, Kohlmann, & Klein-Heßling, 2006, 2018). The original German version of the SSKJ questionnaire focuses on daily stress among children and adolescents and was based on the stress and coping framework by Lazarus and Folkman (1984). This transactional stress model emphasizes both the individuals’ appraisal of situations and coping efforts in response to stressful situations. Thus, what one individual experiences as taxing or exceeding resources, another may perceive as less stressful or even irrelevant. When experiencing stress, the ways children and adolescents cope with stressors influence the impact stress has on symptoms, adjustment and health. Accordingly, the SSKJ questionnaire has three sections that cover these different aspects of stress: (1) stress vulnerability in everyday situations (e.g. ‘Imagine that you had a big argument with a friend. How much stress do you have?’); (2) coping strategies (i.e. seeking social support, problem solving, avoidant coping, palliative emotion regulation, and anger-related emotion regulation); and (3) stress-related symptoms and well-being. Meanwhile, for the SSKJ coping scales (Part 2), several language versions are available that have been evaluated for their measurement invariance (Eschenbeck et al., 2020; Eschenbeck, Heim-Dreger, Tasdaban, Lohaus, & Kohlmann, 2012; Weis & Heine, 2020). This study refers to the Stress-Symptom and Well-Being Scales (Part 3).

The SSKJ Stress-Symptom and Well-Being Scales capture somatic symptoms (e.g. headaches, stomach aches), psychological symptoms (including the three central facets anger, sadness, and anxiety), and well-being (in each case, referring to the previous week). With regard to the development of the scales, previously only somatic and psychological symptoms (i.e. anger, sadness, anxiety) were assessed (Lohaus et al., 2006). In order to focus not only on negative effects of stressful experiences, feelings of well-being (i.e. being happy, gleeful, cheerful, in a good temper) were added in a revised version of the SSKJ questionnaire (Lohaus, Eschenbeck, Kohlmann, & Klein-Heßling, 2018). The SSKJ Stress-Symptom and Well-Being Scales are available in several languages. To date, however, only the German version has been evaluated (Lohaus et al., 2006, 2018). The five-factor structure (somatic symptoms, anger, sadness, anxiety, and well-being) provided an acceptable fit. Internal consistency (Cronbach’s α from .71 to .81/.67 to .79 for the SSKJ 3-8/SSKJ 3-8 R, mean α = .76/.72) and test-retest reliability (time period 1–3 weeks; *r*_tt_ from .56 to .70/.61 to .66 for the for the SSKJ 3-8/SSKJ 3-8 R, mean *r*_tt_ = .63/.64) were mostly satisfying. There was also evidence for convergent validity for the SSKJ Stress-Symptom Scales: increased symptom reporting was associated with decreased health-related quality of life, increased behavioral problems and increased anger-related emotion regulation (Eschenbeck et al., 2012; Lohaus et al., 2006, 2018). Regarding gender differences, comparable to other symptom scales (for the HBSC checklist: e.g. Haugland, Wold, Stevenson, Aaroe, & Woynarowska, 2001; Kelly, Molcho, Doyle, & Gabhainn, 2010; Torsheim et al., 2006; for the PANAS: e.g. Ciucci et al., 2017), in general, girls reported more stress-related symptoms than boys, except for the subscale Anger which indicated no gender differences (Lohaus et al., 2018).

Recent studies (e.g. Guse & van Zyl, 2018; Hagquist, Due, Torsheim, & Välimaa, 2019) have addressed cross-cultural comparisons (among countries, ethnicities, or language versions of questionnaires) as a further influencing factor of symptom reports. As a prerequisite for these comparisons, evidence on measurement equivalence is demanded (Stevanovic et al., 2017). Overall, however, in their review on childhood psychopathological symptom scales, Stevanovic et al. (2017) clearly showed only limited testing for measurement invariance across cultural groups, countries, or language versions of the questionnaires.

Thus, to be able to interpret group differences, questionnaires used should measure in an invariant way across the groups (e.g. gender or language versions). It is unclear, however, whether the SSKJ Stress-Symptom and Well-Being Scales measure in a similar manner across girls and boys or different languages. Therefore, the first aim of the present study was to analyse whether measurement invariance of the SSKJ Stress-Symptom and Well-Being Scales manifests for five newly-developed language versions: English, French, Russian, Spanish, and Ukrainian. For this, the study covers various countries, including Western regions (France, Germany, Great Britain, Ireland, Australia, the United States), Southern regions (Dominican Republic) and Eastern regions (Ukraine, Russia). The availability of an evaluated stress-symptom measure for children and adolescents from Western and Eastern regions, speaking different languages, is of high importance for cross-country studies on stress and well-being. Based on the original German SSKJ Stress-Symptom and Well-Being Scales (Lohaus et al., 2018) and on our study showing partial metric invariance for the SSKJ Coping Scales (Eschenbeck et al., 2020), we expected that a five-factor model would provide an acceptable fit and that at least partial measurement invariance across the language versions of the SSKJ Stress-Symptom and Well-Being Scales would be confirmed. Second, in terms of convergent validity, associations of the SSKJ Stress-Symptom and Well-Being Scales with the SDQ as an indicator of mental health and behavioral problems will be examined. Across the different language versions, we generally expected positive associations between stress-symptoms and psychological maladjustment (Lohaus et al., 2018). Third, with regard to gender differences, it was also hypothesized that at least partial measurement invariance across gender would be established. When measurement equivalence across groups was confirmed, we sought to determine whether gender differences in symptom reports (girls greater than boys) were consistent across the different languages. Thus, we evaluated gender differences in the experience of stress-symptoms and well-being among children and adolescents speaking different languages.

## Method

### Participants

Participants were 5,347 children and adolescents recruited from elementary schools and high schools in various countries. Questionnaires in which items remained unanswered were excluded from the analyses. Thus, the final sample consisted of 5,227 children and adolescents from Germany (*n* = 3,150; i.e. German-speaking sample), France (*n* = 318; French sample), Russia (*n* = 376; Russian sample), the Dominican Republic (*n* = 207; Spanish sample), Ukraine (*n *= 492; Ukrainian sample), and several English-speaking countries such as Australia, Great Britain, Ireland, and the USA (English sample: *n* = 684). Gender and age demographics for the different language subsamples are shown in Table 1. Boys were overrepresented in the English sample, *Chi*^2^ (5, *n* = 5216) = 60.76, *p* < .001. In terms of age (total sample: *M* = 11.53 years, *SD* = 1.91, median = 12, range 7–18 years), the language sub-samples differed, *F* (5, 5207) = 120.31, *p* < .001, Eta^2^ = 104. Children were youngest in the German and Spanish samples and oldest in the Ukrainian sample.

**Table 1.** Sample Description and Means (M), Standard Deviations (SD) and Internal Consistencies (Cronbach’s α, McDonald's ω) for the Different Language Versions of the SSKJ 3–8 Stress-Symptom and Well-Being Scales.

	German (*n* = 3,150)	English (*n* = 684)	French (*n* = 318)	Russian (*n* = 376)	Spanish (*n* = 207)	Ukrainian (*n* = 492)
Age						
*M (SD)*	11.14 (1.83)	11.54 (1.82)	12.02 (1.49)	12.46 (1.14)	11.46 (2.21)	13.03 (2.08)
Gender, *n (%)*						
Male	1,563 (49.7%)	447 (65.5%)	165 (52.1%)	176 (47.1%)	108 (52.7%)	252 (51.3%)
Female	1,584 (50.3%)	235 (34.5%)	152 (47.9%)	198 (52.9%)	97 (46.9%)	239 (48.7%)
SOM, *M (SD)*	9.87 (2.79)	10.64 (2.90)	9.99 (2.80)	9.87 (2.67)	10.22 (2.69)	9.33 (2.46)
Internal consistency	.67 (.69)	.68 (.70)	.67 (.69)	.63 (.66)	.59 (.59)	.63 (.64)
ANG, *M (SD)*	7.38 (2.41)	7.90 (2.34)	7.33 (2.33)	6.80 (2.45)	6.64 (2.00)	6.99 (2.42)
Internal consistency	.79 (.80)	.78 (.80)	.77 (.80)	.82 (.81)	.61 (.65)	.83 (.83)
SAD, *M (SD)*	6.50 (2.19)	6.88 (2.27)	6.70 (2.40)	7.37 (2.42)	6.81 (2.22)	6.53 (2.04)
Internal consistency	.73 (.74)	.78 (.78)	.78 (.81)	.75 (.75)	.62 (.64)	.69 (.70)
ANX, *M (SD)*	7.58 (2.20)	8.10 (1.79)	7.83 (2.46)	7.60 (2.51)	7.04 (1.98)	7.03 (2.11)
Internal consistency	.68 (.68)	.42/.68* (.46/.62)	.75 (.69)	.79 (.78)	.51/.62* (.50/.56)	.66 (.67)
WELL, *M (SD)*	10.78 (1.76)	10.42 (1.76)	10.73 (1.66)	10.90 (1.75)	10.96 (1.74)	10.51 (1.99)
Internal consistency	.72 (.72)	.66 (.66)	.63 (.61)	.75 (.73)	.75 (.76)	.79 (.79)

Note*.* SOM = Somatic Symptoms (6 items), ANG = Anger (4 items), SAD = Sadness (4 items), ANX = Anxiety (4 items/3 items: without Item 15, Spearman-Brown adjusted*), WELL = Well-Being (4 items). Internal consistency estimates: Cronbach's *α* (McDonald’s ω). Instruction: ‘How did you feel in the last week?’ 3-point rating scale: not once/once/many times.

Missing information on age/gender (number of cases): German (2/3), English (1/2), French (0/1), Russian (13/2), Spanish (0/2), Ukrainian (1/1), Total sample (17/9).

### Procedure

Pupils were recruited through public schools from both urban and rural areas in the regions of Baden-Wuerttemberg and North Rhine-Westphalia (Germany; 49 schools); Auvergne-Rhone-Alpes, Normandy, and Paris (France; four schools); Irkutsk (Russia; one school); Jarabacoa (the Dominican Republic; one school); the greater Odessa area (Ukraine; five schools); the Sydney area (Australia; one school); Pontypridd and Slough (Great Britain; three schools); the counties Cork, Longford, and Westmeath (Ireland; three schools); and Charlotte (USA; one school). Most of the data collection took place between 2015 and 2017, with the exception of one French school (*n* = 100) collected in 2012, and data from the school in the USA (*n* = 63), collected in 2014. Informed consent was given by the pupils and their parents prior to the start of the study. Participants completed the questionnaires in their classes. The survey lasted about one school lesson and was generally carried out by a trained supervisor or teacher. Participants did not receive any credits for participation. The study was conducted according to the ethical guidelines of the Declaration of Helsinki. Human participants’ protection was approved by the University of Education Weingarten, Germany (the institution at which the corresponding author was when the study started).

### Measures

#### Stress-Symptom and Well-Being Scales

The Stress-Symptom and Well-Being Scales from the *Stress and Coping Questionnaire for Children and Adolescents* (SSKJ 3–8 R; Lohaus et al., 2018) is a 22-item self-report instrument originally developed in the German language. It assesses stress symptomatology and well-being experienced in the last week with five subscales: Somatic Symptoms, Anger, Sadness, Anxiety, and Well-Being. Participants use a 3-point rating scale to mark responses ranging from *never* = 1 to *several times* = 3. Scale descriptives and internal consistencies (Cronbach’s *α*, McDonald’s ω) are reported in Table 1. The original German version of the SSKJ Stress-Symptom and Well-Being Scales was translated into five languages (English, French, Russian, Spanish, and Ukrainian). Separately for each language version, the translation was developed using the translation-back-translation procedure recommended by Brislin (1970). The original German questionnaire was translated into the target language by a translator who was a native speaker of the respective language (English, French, Russian, or Ukrainian) or a professional translator (Spanish, with knowledge of the language used in the Dominican Republic). The back-translation into the German version was done by a second translator who was unaware of the original questionnaire. The second translator was bilingual (German and one of the following languages English, French, or Russian) or a professional translator (Spanish, Ukrainian) with knowledge of the respective culture. During the translation, attention was paid to a language suitable for children and adolescents. Next, we compared the back-translated German version with the original version. Together with the translators, discrepancies were discussed until agreement on a common version was reached. Finally, native speakers (mainly elementary school or high school teachers in the respective countries), who were not involved in the translation process, reviewed the adapted questionnaire. Short versions of the items can be found in Supplementary Table 1. In addition, coping strategies (Lohaus et al., 2018) were assessed. These data were not taken into account in the present study. Results for the coping scales not related to the present study were reported in Eschenbeck et al. (2020).

#### Strengths and Difficulties Questionnaire

The Strengths and Difficulties Questionnaire (SDQ; Goodman, 1997) was used as a brief 25-item, self-report screening questionnaire for mental health and behavioral problems (referring to the last 6 months) in the language versions English, French, Russian, Spanish, and Ukrainian (available under https://www.sdqinfo.org). However, it was not administered in all English and French schools. For this study, we used the subscale Prosocial Behavior (5 items) and the Total Difficulties Score that covers four subscales of mental health problems (i.e. emotional problems, conduct problems, hyperactivity/inattention, peer relationship problems; 20 items, 3-point rating scale from *not true* = 0 to *certainly true* = 2). Internal consistencies (Cronbach’s *α*) are reported in Table 2. Items that were only weakly related (*r* < .10) to their corresponding scale were excluded. This concerned the Total Difficulties Score of both the Russian version (items 7 and 23 were excluded; Cronbach’s *α* = .78 for the original 20-item version; correlation between the 20- and 18-item total scores *r* = .99) and the Ukrainian version (items 2, 7, 10, 11, 23 were excluded; *α* = .58 for the original 20-item version; correlation between the 20- and 15-item total scores *r = *.94).

**Table 2.** Descriptive Statistics and Correlations With Mental Health and Behavioral Problems (Separately for the Language Versions).

				*r*				
				SSKJ Stress-Symptoms and Well-Being				
	SDQ	*M* (*SD*)	*α*	SOM	ANG	SAD	ANX	WELL
English (*n* = 596)	Prosocial Behavior	1.45 (0.41)	.68	−.02	−.13**	−.02	−.01	.23***
	Total Difficulties Score	0.62 (0.31)	.81	.49***	.54***	.53***	.49***	−.27***
French (*n* = 145)	Prosocial Behavior	1.52 (0.36)	.63	−.02	−.06	.20*	.14	.28**
	Total Difficulties Score	0.59 (0.29)	.77	.36***	.40***	.27**	.26**	−.24**
Russian (*n* = 373)	Prosocial Behavior	1.45 (0.42)	.70	−.14**	−.20***	−.06	−.04	.36***
	Total Difficulties Score^a^	0.56 (0.32)	.80	.46***	.52***	.52***	.49***	−.30***
Spanish (*n* = 207)	Prosocial Behavior	1.34 (0.50)	.69	−.07	−.08	−.05	.08	.44***
	Total Difficulties Score	0.67 (0.30)	.72	.38***	.36***	.43***	.26***	−.29***
Ukrainian (*n* = 462)	Prosocial Behavior	1.37 (0.47)	.70	−.04	−.15***	−.08	−.09	.30***
	Total Difficulties Score^b^	0.56 (0.32)	.69	.27***	.42***	.37***	.38***	−.33***

Note*.* SDQ = Strengths and Difficulties Questionnaire (range: 0–2), SSKJ 3–8 = Stress and Coping Questionnaire for Children and Adolescents, SOM = Somatic Symptoms, ANG = Anger, SAD = Sadness, ANX = Anxiety, WELL = Well-Being. *α* = Cronbach's *α*.

^a^ without items 7, 23, ^b^without items 2, 7, 10, 11, 23.

* *p* < .05, ** *p* < .01, *** *p* < .001.

### Statistical analyses

All analyses were carried out using the statistical software R (version 3.5.2 for Windows; R Core Team, 2016). Overall, our analysis procedure included relevant steps for scale validation outlined in Dima (2018), except for investigations of non-parametric item response theory. Since the SSKJ Stress-Symptom and Well-Being Scales are measured using 3-point ordinally scaled indicator variables, we specified the WLSMV estimator for complete data and used the lavaan package (version 0.6-3; Rosseel, 2012) for model estimation. Measurement invariance for the five language versions was assessed with reference to Brown (2015). We first established *configural invariance* by testing whether the factorial structure (i.e. the pattern of freely-estimated and restricted parameters) established for the original German SSKJ Stress-Symptom and Well-Being Scales (Lohaus et al., 2018) can be replicated for each language version, as indicated by a good model fit. We then tested for invariance by means of two strategies involving multi-group confirmatory factor analysis. In our first strategy, each multi-group analysis consisted of two groups; one non-German sample and a corresponding German subsample matched to the non-German sample according to gender and age (with a sample size not greater than three times the non-German sample). In our second strategy, multi-group analyses consisted of six groups using the full sample of each language version as one group. We tested for *metric invariance* (equal factor loadings across groups) by fitting (a) a model with freely-estimated factor loadings, and (b) a model with factor loadings restricted to be equal between the groups. We evaluated if the model with equal loadings, provided a comparably good fit to the model with group-specific loadings. If metric invariance was established, we further restricted the thresholds of the indicators in additional multi-group analyses in order to test for *scalar invariance*. These analyses evaluated if the indicator-specific thresholds which determine the observed indicator values can be considered equal between groups. Since the chi-square test statistic and, in consequence, the likelihood-ratio test, are sensitive to sample size, fit indices were used for evaluating the model fit (cf. Beauducel & Wittmann, 2005; Hu & Bentler, 1999). We calculated the comparative fit index (CFI) and the Tucker-Lewis index (TLI) as incremental measures (values ≥ .95 indicate a good fit), the root mean square error of approximation (RMSEA; values ≤ .06 indicate good fit) and the standardized root mean squared residual (SRMR; values ≤ .08 indicate good fit) as absolute measures of fit. Cheung and Rensvold (2002) proposed the change in fit indices in increasingly restrictive models as a criterion for significant deteriorations of a restrictive model (e.g. metric invariance) compared to a less restrictive model (e.g. configural invariance). As suggested by Meade, Johnson, and Braddy (2008), we evaluated changes in values for the comparative fit index (ΔCFI) and McDonald’s noncentrality index (ΔNCI). Cheung and Rensvold (2002) suggest values of ΔCFI > .01 and ΔNCI > .02 to indicate a substantial change in model fit, while Meade et al. (2008) postulated stricter limits (ΔCFI > .002 and ΔNCI > .0075 for the proposed factorial structure of five factors with 22 indicator variables).

Additionally, for each language version, we tested if the factor structure is invariant regarding the participants’ gender, using the same testing strategy described above. In these analyses, the groups for the multi-group analyses consisted of either all male or female children of each language version. Metric invariance hence evaluated whether factor loadings can be considered equal between boys and girls of a given language while scalar invariance evaluated if, additional to factor loadings, indicator thresholds are equal between boys and girls.

## Results

### Configural invariance of the Stress-Symptom and Well-Being Scales

**Figure 1.**
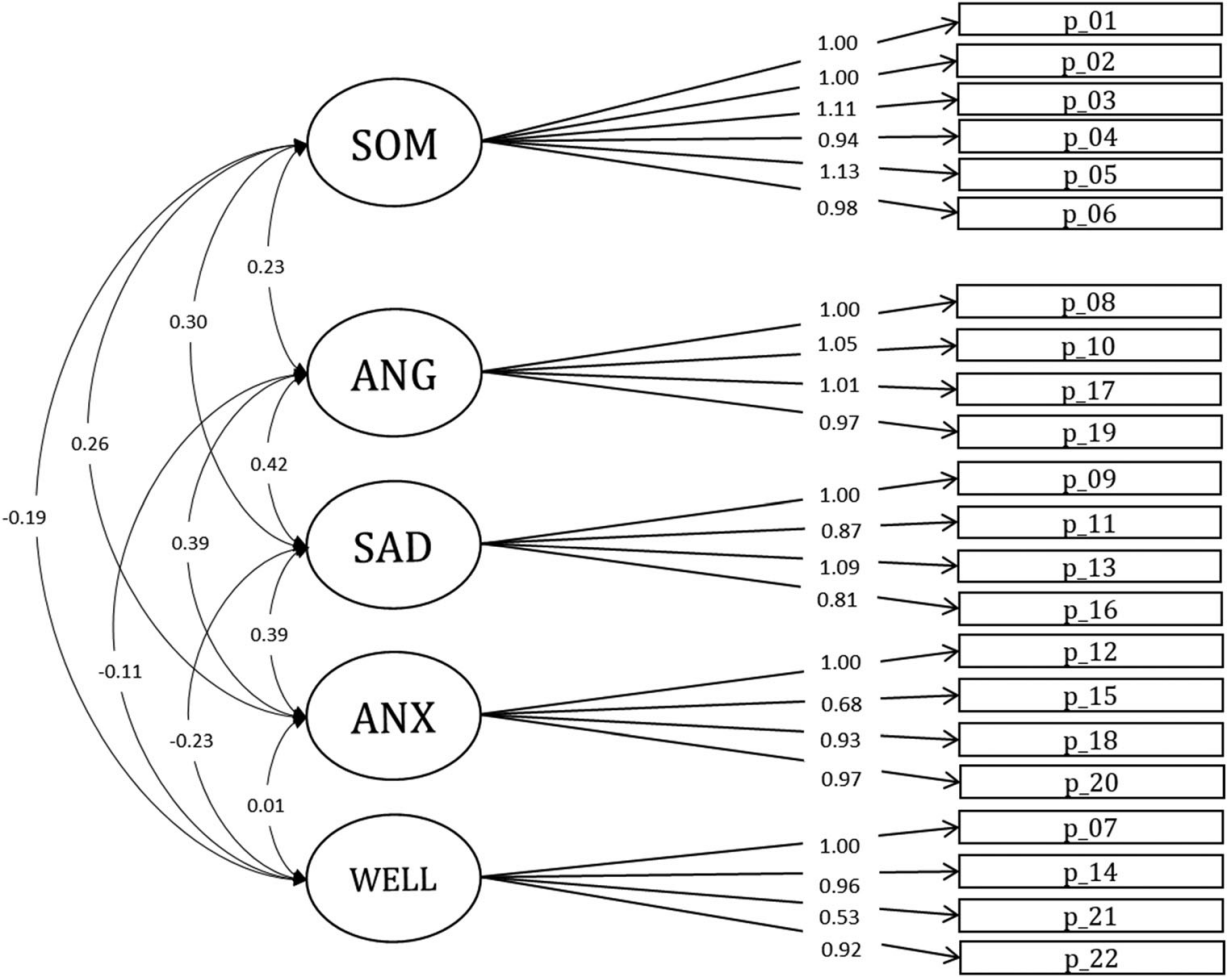
Five-Factor Structure of the SSKJ 3–8 Stress-Symptom and Well-Being Scales (German Version). Note. SOM = Somatic Symptoms, ANG = Anger, SAD = Sadness, ANX = Anxiety, WELL = Well-Being.

The model of the German version of the SSKJ Stress-Symptom and Well-Being Scales with the five factors somatic symptoms, anger, sadness, anxiety, and well-being is shown in Figure 1. Factor loadings for the WLSMV estimation ranged from 0.53–1.13. In terms of the model fit indices, results for the five-factor model of the German sample are mixed (see Table 3); while the SRMR and RMSEA suggest good fit, CFI and TLI indicate mediocre fit to the data. The inter-correlations for the five factors are relatively high, especially between the emotional symptom scales of Anger, Sadness, and Anxiety (*r *≥ .70 for *r*_anger⨯sadness_ and *r*_anxiety⨯sadness_, see Supplementary Table 2). The application of this five-factor model to the five non-German language versions of the SSKJ Stress-Symptom and Well-Being Scales also provided mixed results: The CFI and TLI showed values of .90 or higher for all but the English language version and .95 or higher for the Spanish and Ukrainian sample. The RMSEA coefficients showed values of .06 or less for three of the five versions (Russian, Spanish, and Ukrainian), the SRMR showed values of .08 or less for four versions (English, Russian, Spanish, and Ukrainian). Ranges in loadings were 0.59–2.15 for the English language version, 0.37–1.30 for the French version, 0.58–1.18 for the Russian version, 0.28–1.39 for the Spanish version, and 0.65–1.19 for the Ukrainian version. In summary, ranges were smallest for the German, French and Spanish sample, and largest for the English one.

**Table 3.** Model Fit Indices^a^ of the SSKJ 3–8 Stress-Symptom and Well-Being Scales for the Six Language Versions.

	German	English	French	Russian	Spanish	Ukrainian
*N*	3,150	684	318	376	207	492
Chi²-value	2116.862	1018.725	480.983	409.684	198.233	242.271
*df*	199	199	199	199	199	199
CFI	.939	.899	.934	.949	1.000	.977
TLI	.929	.882	.923	.941	1.001	.973
RMSEA (90% CI)	.055 (.052; .057)	.078 (.073; .082)	.067 (.059; .074)	.063 (.056; .070)	.000 (.000; .003)	.038 (.031; .045)
SRMR	.061	.080	.091	.081	.070	.056

Note*.* ^a^Robust results using WLSMV estimation. CFI = Comparative Fit Index, TLI = Tucker-Lewis Index, RMSEA = Root Mean Square Error of Approximation and 90 % confidence interval, SRMR = Standardized Root Mean Square Residual.

Regarding the correlational structure (Supplementary Table 2), high correlations (*r *≥ .70) were found between somatic symptoms and emotional symptoms as well as within the emotional stress-symptoms. In general, symptom factors correlated negatively with well-being, and the strength of these associations was smaller than the associations within the symptom factors. Especially for the Ukrainian version and the Russian version, factors correlated highly (*r *≥ .90 for *r*_anxiety⨯sadness_ and *r*_anxiety⨯anger_, see supplementary Table 2). A subsequent explorative factor analysis (Promax rotation; Eigenvalues of components were chosen according to principal axis factor analysis) for both language versions separately indicated four factors each with eigenvalues > 1 (Ukrainian: λ = 6.64, 2.34, 1.34, 1.07, 0.94, 0.91, … ; Russian: λ = 7.01, 2.50, 1.43, 1.25, 1.01, 0.91, …). Yet, additional fit analyses using reasonable alternative models with 3-factor, 4-factor, and 5-factor solutions yielded no model with superior fit to the proposed 5-factor model of the SSKJ Stress-Symptom and Well-Being Scales. Therefore, to ensure comparability, the five-factor structure was retained for subsequent analyses. In sum, configural invariance (the same five-factor structure across different language versions) can cautiously be assumed for the five additional language versions of the SSKJ Stress-Symptom and Well-Being Scales (English, French, Russian, Spanish, and Ukrainian). Therefore, metric invariance was tested.

### Metric and scalar invariance of the stress-Symptom and Well-Being Scales for the different language versions

Following our first testing strategy, the invariance of the factor loadings was evaluated by five multi-group CFAs. For each of these five tests, a subsample of the German sample was randomly drawn which matched the distribution of age and gender in the respective comparison sample (English, French, Russian, Spanish, or Ukrainian). The fit statistics for the invariance tests are reported in Table 4. Results of the comparisons showed mediocre to good model fits for the configural invariance models. Restricting loadings to be equal, the analyses support metric invariance of the SSKJ Stress-Symptom and Well-Being Scales according to the proposed values by Cheung and Rensvold (2002) for four language versions (French, Russian, Spanish, and Ukrainian). Additionally, metric invariance was also supported by the stricter limits set by Meade et al. (2008) for the Ukrainian language version. Therefore, additional analyses regarding scalar invariance (i.e. the invariance of indicator intercepts) were performed for four language versions (i.e. for all but the English one).

**Table 4.** Tests^a^ of Metric Invariance of the SSKJ 3–8 Stress-Symptom and Well-Being Scales Across Different Language Versions.

	Chi^2^	*df*	RMSEA	CFI	ΔCFI	NCI	ΔNCI	Decision
German – English								
Configural invariance	2353.012	398	.060	.933		.698		
Metric invariance	3079.261	415	.069	.909	.024	.613	.085	Reject
Scalar invariance	3472.443	432	.072	.896	.013	.572	.041	-
German – French								
Configural invariance	1180.207	398	.056	.941		.734		
Metric invariance	1300.991	415	.058	.933	.008^b^	.705	.029	Accept
Scalar invariance	1516.302	432	.063	.918	.015	.652	.053	Reject
German – Russian								
Configural invariance	1305.213	398	.056	.940		.730		
Metric invariance	1264.872	415	.053	.944	.004^b^	.745	.015^b^	Accept
Scalar invariance	1696.433	432	.064	.917	.027	.645	.100	Reject
German – Spanish								
Configural invariance	612.235	398	.036	.965		.876		
Metric invariance	660.507	415	.038	.959	.005^b^	.860	.017^b^	Accept
Scalar invariance	754.260	432	.043	.947	.013	.820	.040	Reject
German – Ukrainian								
Configural invariance	1438.36	398	.052	.952		.767		
Metric invariance	1460.78	415	.051	.952	.000^b,c^	.766	.001^b,c^	Accept
Scalar invariance	1820.695	432	.057	.936	.016	.702	.064	Reject
Full sample – six languages								
Configural invariance	4276.925	1194	.054	.944		.745		
Metric invariance	5260.595	1279	.060	.927	.017	.683	.062	Reject
Partial metric invariance	4290.702	1274	.052	.945	−.001^b,c^	.749	−.004^b,c^	(Accept)

Note*.* ^a^robust results using WLSMV estimation; CFI = scaled Comparative Fit Index, NCI = McDonald’s noncentrality index, RMSEA = scaled Root Mean Square Error of Approximation, Δ = scaled change in the parameter.

Sample sizes for the matched data: German (*n* = 2,043) – English (*n* = 681), German (*n* = 951) – French (*n* = 317), German (*n* = 1,083) – Russian (*n* = 361), German (*n* = 609) – Spanish (*n* = 203), German (*n* = 1,470) – Ukrainian (*n* = 490). Sample sizes for the unmatched data: German (*n* = 3150), English (*n* = 684), French (*n* = 318), Russian (*n* = 376), Spanish (*n* = 207), Ukrainian (*n* = 492). ^b^invariance indicated by ΔCFI ≤ .01 and/or ΔNCI ≤ .02 (Cheung & Rensvold, 2002); ^c^invariance indicated by ΔCFI ≤ .002 and/or ΔNCI ≤ .0075 (Meade et al., 2008).

Testing for scalar invariance (i.e. the same factorial structure, loadings, and thresholds) revealed that none of the four models’ changes in fit remained within the admissible range. Therefore, scalar invariance was not supported for the different language versions.

Following our second testing strategy, the invariance of the factor loadings was evaluated by one multi-group CFA, using each language sample as one group. The fit statistics for the invariance tests are reported in the bottom rows of Table 4. Results show an almost acceptable model fit for the configural invariance model. Restricting loadings to be equal leads to decreased fit, such that full metric invariance of the SSKJ Stress-Symptom and Well-Being Scales was not supported. Following an evaluation of the Lagrange Multiplier Test scores, we released the restriction on the loading parameter of Item 15 of the Anxiety subscale, and the resulting model fits marginally better than the configural invariance model (ΔCFI = −.001, ΔNCI = −.004). Hence, partial metric invariance can be achieved by allowing the loading of one item to vary across language versions.^1^

### Correlations with mental health and behavioral problems (SDQ)

Table 2 presents the correlations between the SSKJ Stress-Symptom and Well-Being Scales and the SDQ subscale Prosocial Behavior and the Total Difficulties Score. As expected, Somatic Symptoms, Anger, Sadness and Anxiety were positively correlated with the SDQ Total Difficulties Score (*r*s from .26 to .54 across language versions). The correlations with Prosocial Behavior were low and largely not significant (*r*s < .20). Well-being was positively associated with Prosocial Behavior (.23 to .44), and was negatively associated with the SDQ Total Difficulties Score (−.24 to −.33).

### Metric and scalar invariance of the Stress-Symptom and Well-Being Scales for boys and girls

The invariance of the factor loadings between boys and girls was evaluated by six multi-group CFAs (for each language separately). The fit statistics for the invariance tests are reported in Table 5. Results of the comparisons showed mediocre to very good model fits for the configural invariance models. Restricting loadings to be equal between boys and girls, all but the Spanish language analysis corroborated metric invariance according to Cheung and Rensvold (2002) with ΔCFA ≤ .01 and additionally ΔNCI ≤ .02 for the German, French, and Ukrainian version. Hence, the five language versions (German, English, French, Russian, and Ukrainian) were further tested for scalar invariance.

**Table 5.** Tests^a^ of Metric Invariance of the SSKJ 3–8 Stress-Symptom and Well-Being Scales Between Boys and Girls for the Different Language Versions.

	Chi^2^	*df*	RMSEA	CFI	ΔCFI	NCI	ΔNCI	Decision
German: Boys-Girls								
Configural invariance	2245.245	398	.054	.941		.746		
Metric invariance	2135.594	415	.051	.945	.004^b^	.761	.015^b^	Accept
Scalar invariance	2376.01	432	.053	.938	.007^b^	.734	.027	Accept
English: Boys-Girls								
Configural invariance	1121.25	398	.073	.906		.588		
Metric invariance	1081.427	415	.069	.913	.007^b^	.613	.025	Accept
Scalar invariance	1152.167	432	.070	.906	.007^b^	.589	.024	Accept
French: Boys-Girls^d^								
Configural invariance	641.640	398	.062	.936		.680		
Metric invariance	641.175	415	.059	.941	.005^b^	.699	.019^b^	Accept
Scalar invariance	675.873	432	.060	.936	.005^b^	.680	.019^b^	Accept
Russian: Boys-Girls								
Configural invariance	662.612	398	.060	.951		.701		
Metric invariance	648.227	415	.055	.957	.006^b^	.732	.030	Accept
Scalar invariance	694.575	432	.057	.952	.005^b^	.703	.028	Accept
Spanish: Boys-Girls^d^								
Configural invariance	407.811	398	.016	.992		.976		
Metric invariance	443.004	415	.026	.977	.015	.934	.043	Reject
Scalar invariance	449.788	432	.020	.985	.008	.957	.024	-
Ukrainian: Boys-Girls								
Configural invariance	513.068	398	.034	.981		.889		
Metric invariance	512.288	415	.031	.984	.003^b^	.905	.016^b^	Accept
Scalar invariance	565.476	432	.036	.978	.006^b^	.873	.033	Accept

Note*.* ^a^robust results using WLSMV estimation; CFI = scaled Comparative Fit Index, NCI = McDonald’s noncentrality index, RMSEA = scaled Root Mean Square Error of Approximation, Δ = scaled change in the parameter.

^b^ invariance indicated by ΔCFI ≤ .01 and/or ΔNCI ≤ .02 (Cheung & Rensvold, 2002); ^c^invariance indicated by ΔCFI ≤ .002 and/or ΔNCI ≤ .0075 (Meade et al., 2008). ^d^Estimation issues emerged for the French sample (one negative variance estimate) and the Spanish sample (two correlation estimates > 1.00). Additional restrictions to admissible values have been set for sensible model estimation.

As shown in Table 5, scalar invariance between boys and girls (i.e. the same factorial structure, loadings, and thresholds) was supported for the five languages (English, German, French, Russian, and Ukrainian) with ΔCFA ≤ .01 and additionally ΔNCI ≤ .02 for the French version.

### Mean differences between boys and girls

To examine the effects of gender on stress-symptoms and well-being across the five language versions for which scalar invariance was confirmed (English, German, French, Russian, and Ukrainian), for each subscale (Somatic Symptoms, Anger, Sadness, Anxiety, and Well-Being) we conducted a covariance analysis (ANCOVA) with gender as the between-subject factor and age as control variable. Results are reported in Table 6. Girls scored higher on Somatic Symptoms, Sadness, and Anxiety (each except for the English- and Ukrainian-speaking samples). Within the French sample, girls reported higher values on Anger, too. For Well-Being, in two samples (German and Ukrainian), there were gender differences in favor of the girls. Overall, the effect sizes were rather small (partial η² ≤ .055; Cohen, 1988).

**Table 6.** Mean Differences Between Boys and Girls.

	German (*n* = 3,150)		English (*n* = 684)		French (*n* = 318)		Russian (*n* = 376)		Ukrainian (*n* = 492)	
Subscales	*M (SD)*	*F*-value Part *Eta^2^*	*M (SD)*	*F*-value Part *Eta^2^*	*M (SD)*	*F*-value Part *Eta^2^*	*M (SD)*	*F*-value Part *Eta^2^*	*M (SD)*	*F*-value Part *Eta^2^*
SOM										
Male	9.43 (2.66)	84.09***	10.59 (2.88)	0.04	9.39 (2.51)	15.62***	9.31 (2.41)	13.28**	9.04 (2.41)	6.51
Female	10.31 (2.85)	.026	10.70 (2.95)	.000	10.61 (2.95)	.047	10.31 (2.83)	.036	9.62 (2.49)	.013
ANG										
Male	7.35 (2.44)	0.77	7.93 (2.34)	0.11	6.95 (2.26)	9.79*	6.67 (2.44)	0.41	6.98 (2.27)	0.04
Female	7.40 (2.38)	.000	7.84 (2.34)	.000	7.76 (2.34)	.030	6.81 (2.40)	.001	7.00 (2.58)	.000
SAD										
Male	6.07 (2.00)	120.36***	6.91 (2.28)	0.99	6.18 (2.20)	18.18***	6.83 (2.29)	12.41**	6.31 (1.97)	5.48
Female	6.91 (2.29)	.037	6.82 (2.24)	.001	7.30 (2.47)	.055	7.68 (2.40)	.034	6.75 (2.10)	.011
ANX										
Male	7.42 (2.21)	18.60***	8.17 (1.83)	0.71	7.21 (2.33)	23.19***	7.18 (2.41)	6.71*	7.06 (2.09)	0.07
Female	7.74 (2.18)	.006	7.95 (1.70)	.001	8.49 (2.43)	.069	7.84 (2.53)	.018	7.00 (2.14)	.000
WELL										
Male	10.67 (1.86)	11.23**	10.44 (1.72)	0.03	10.58 (1.77)	2.70	10.69 (1.89)	4.57	10.28 (2.15)	7.58*
Female	10.88 (1.64)	.004	10.38 (1.81)	.000	10.89 (1.51)	.009	11.08 (1.64)	.013	10.74 (1.77)	.015

Notes*.* SOM = Somatic Symptoms (6 items), ANG = Anger (4 items), SAD = Sadness (4 items), ANX = Anxiety (4 items), WELL = Well-Being (4 items). Instruction: ‘How did you feel in the last week?’ 3-point rating scale: not once/once/many times.

Missing information on age/gender (number of cases): German (2/3), English (1/2), French (0/1), Russian (13/2), Ukrainian (1/1).

*** *p* < .001, ** *p* < .01, * *p* < .05 (Bonferroni adjusted).

## Discussion

The present study aimed to investigate the factorial structure and the measurement invariance of the SSKJ Stress-Symptom and Well-Being Scales across gender and different language versions (English, French, German, Russian, Spanish, and Ukrainian) within children and adolescents from various countries, including Western, Southern, and Eastern regions. Moreover, we studied associations with the SDQ as an indicator of mental health and behavioral problems and gender differences. Overall, the SSKJ Stress-Symptom and Well-Being Scales showed psychometric properties that ranged from acceptable to good. The five-factor structure of the original German version (Lohaus et al., 2018), with somatic stress-symptoms, anger, sadness, anxiety, and well-being, was replicated for boys and girls as well as for the different language versions. For the Ukrainian and the Russian version, however, the factorial structure could be simplified. Exploratory analyses pointed to a three or four factor structure with less differentiation within the emotional stress-symptoms. However, additional fit analyses did not confirm a simpler model with superior fit to the proposed five factors. Therefore, to allow for consistent comparisons across the different languages, the five-factor structure was retained for subsequent analyses. Except for this, however, the factorial structure was equal to the original dimensionality of the questionnaire, indicating that independent of the child’s gender or language used, children and young adolescents largely interpreted the items of the four stress-symptom scales and the well-being scale in a similar way. As a consequence, the instrument allows for consistent comparisons across the different languages and for differentiated insights into the somatic and emotional stress-symptoms experienced by children and adolescents.

Moreover, the results of multi-group CFAs supported *metric invariance* (weak invariance), implying that factor loadings are equal for the different languages and between boys and girls (with one exception each). This means that stress-symptoms and well-being measured with the SSKJ have similar meanings across language versions and gender (except for the English version when compared to the German version and for Spanish-speaking boys and girls). Regarding the separate comparisons between the original German version and the different languages, metric invariance was confirmed for the four comparisons, German-French, German-Russian, German-Spanish, and German-Ukrainian, thus including Western (France, Germany), Southern (the Dominican Republic) and Eastern regions (Ukraine, Russia). Scalar invariance (i.e. strong invariance) for the different language versions, however, was not shown. Furthermore, the results supported *partial metric invariance* when analyzing the six language versions together in a full-fledged comparison. Here, the loading of one item of the subscale Anxiety was non-equivalent across the different languages and had to be set free. When excluding the identified item, however, *partial scalar invariance* was met. Taken together, interpretation problems are most likely relevant to the English version and overall to the subscale Anxiety of the SSKJ Stress-Symptom and Well-Being Scales. We therefore recommend improving the translation of Item 15 (‘aufgeregt’, the wording in the original German version). Until then, in cross-country studies, the subscale Anxiety should be considered very carefully or be used with an alternative, less ambiguous anxiety-related adjective for Item 15. The requirement for valid comparisons of latent mean scores across the different languages was not completely met and can be reported as a limitation of the scales. Similar restrictions were documented for the SDQ (with considerably more items being non-invariant across different European countries; Essau et al., 2012; Ortuño-Sierra et al., 2015) or the HBSC checklist with the item ‘sleeping difficulties’ functioning non-equivalent across countries (Ravens-Sieberer et al., 2008).

Regarding gender, *metric invariance* of the Stress-Symptom and Well-Being Scales was confirmed between boys and girls from Germany, France, Russia, Ukraine, and the English-speaking countries. When subsequently tested for *scalar invariance* (i.e. strong invariance), evidence for scalar invariance was shown for these five language versions as well. Thus, within each language mean scores of the SSKJ Stress-Symptom and Well-Being Scales can be meaningfully compared between boys and girls speaking English, German, French, Russian, or Ukrainian. One can only speculate about the non-invariance by gender for the Spanish-speaking sample as previous studies supported measurement invariance across gender for a Spanish stress-response inventory (Ortuño-Sierra, Fonseca-Pedrero, Aritio-Solana, & de Luis, 2016). However, this study was conducted with adolescents living in northern Spain (and thus not like those in the Dominican Republic, as in our study). Moreover, in our investigation, the Spanish-speaking sample was the smallest. Examining gender differences in the symptom reports, the finding that girls reported more symptoms (especially somatic stress-symptoms and sadness) than boys was in accordance with previous studies (e.g. Lohaus et al., 2013), although most effects sizes were small. Somewhat limiting, it should be noted that most previous studies were not genuinely related to stress. For example, in their review Chaplin and Aldao (2013) documented significant but small gender differences with girls showing more internalizing emotions (e.g. sadness, anxiety) compared to boys. On the other hand, the authors reported that, depending on age, middle childhood boys compared to girls of the same age and adolescent girls compared to boys of equal age (similar to the French girls in the present study) showed more externalizing emotions (e.g. anger). Unexpectedly, however, our study did not find gender differences in two (English and Ukrainian) of the five language versions examined. This also differs from HBSC data (Walsh et al., 2020) showing that 15-year-old girls reported more health complaints than boys (including, amongst others, England, Ireland, or the Ukraine). Thus, the overall picture on gender differences in the reported stress-symptoms manifested itself as mixed.

In light of the equivalence of measurement of the SSKJ Stress-Symptom and Well-Being Scales, future studies with larger samples across childhood and adolescence could take into account possible age effects on gender differences and, especially for the English-speaking sample, consider different countries separately (e.g. Torsheim et al., 2006). First of all, however, measurement invariance across age groups (i.e. late childhood, early adolescence, middle adolescence) of the SSKJ Stress-Symptom and Well-Being Scales would have to be checked. Further on, future studies on stress and well-being could explore interaction effects of gender and culture across childhood to adolescence. In this line, cultural dimensions such as collectivism/individualism could be considered in relation to health (e.g. Delvecchio, Li, Liberska, Lis, & Mazzeschi, 2017). Collectivistic cultures promote an interdependent self, emphasise on social dependence, belonging, and social harmony. On the other hand, individualistic cultures promote an independent self, emphasise on personal autonomy, individual initiative, and achievement. Thereby, both cultural orientations can potentially act as risk or protective factor for stress-symptoms and mental health (e.g. Knyazev, Kuznetsova, Savostyanov, & Dorosheva, 2017). Along these lines, it would be interesting to examine the link between daily stress and coping (assessed with the SSKJ questionnaire) and health or illness and whether developmental patterns differ by gender or culture. The associations between the SSKJ Stress-Symptom and Well-Being Scales and indicators of mental health and behavioral problems showed first evidence for convergent validity. However, future prospective studies should elaborate on the association between daily stress and later mental and physical health over time: Stress symptoms and well-being (measured with the SSKJ) would be indicators of early stress responses in everyday life. Mental health problems and symptoms related to psychopathology or physical illness, then, would be more long-term outcomes. Along these lines, a study by Lindholdt et al. (2021) showed that adolescents with high levels of perceived stress were more likely to develop a mental disorder during a 12 months follow-up period. This was beyond the scope of the present paper, at first testing the measurement invariance of the SSKJ Stress-Symptom and Well-Being Scales across gender and different language versions.

There are limitations in our study that must be noted. First, the sample was a convenience sample of children and adolescents from various countries speaking different languages. Within the English version, the sample included four different countries (Australia, Great Britain, Ireland, and the USA), wherein country-specific subsamples were too small to perform separate analyses. However, the samples were always student samples, the access was always via schools, and participants answered questionnaires in their classes. Nevertheless, especially for the Spanish version (as the smallest sample) and the English version (as the most heterogeneous sample in terms of countries), findings should be replicated in larger country-specific samples. Second, regarding internal consistencies, for the SSKJ Stress-Symptom and Well-Being Scales, values greater than .70 were not consistently achieved. Lower values (.60 to .70) were found in particular for the subscales Somatic Symptoms and Anxiety. Reasons might be that the subscales are overall short, the response format is only 3-points, and especially the subscale Somatic Symptoms is heterogeneous in content. For the German version of the SSKJ Stress-Symptom and Well-Being Scales, however, adequate retest reliabilities (*r*_tt_ > .60) were shown (Lohaus et al., 2018). Retest reliabilities for the other language versions are not available and should be examined in further longitudinal studies. Third, for a deeper understanding, additional measures like regional or socio-economic aspects within the countries (e.g. urbanization or socio-economic status) or measures of culture (e.g. collectivism and individualism or acculturation), that may influence children’s and adolescents’ experience of stress and well-being could be included in future research. In this regard, the role of coping strategies and emotion regulation with respect to everyday stressful events, in the context of culture, should also be explored.

To conclude, the SSKJ Stress-Symptom and Well-Being Scales demonstrated partial metric invariance indicating a basic level of comparability of the scales across six different language versions, including English, French, German, Russian, Spanish, and Ukrainian, as was previously documented for the coping scales of the SSKJ questionnaire (Eschenbeck et al., 2020). Thus, associations of stress symptoms and well-being (measured with the SSKJ) with other constructs of interest can be meaningfully compared across different languages. However, cross-country comparisons of mean scores are not recommended. In addition, regarding gender, scalar invariance (strong invariance) was confirmed between boys and girls from Germany, France, Russia, Ukraine and several English-speaking countries. Thus, within these languages, the SSKJ Stress-Symptom and Well-Being Scales measured consistently among boys and girls, allowing for meaningful comparisons of gender differences. Finally, our study indicated that girls reported more stress-related symptoms than boys (except for the English and Ukrainian samples) and provided evidence for associations with indicators of mental health and behavioral problems.

## Data Availability

The data that support the findings of this study are available from the corresponding author upon reasonable request.

## Note

1 Additional analyses that excluded Item 15 of the subscale Anxiety indicated a good model fit for the *configural invariance* model (CFI = .960). Restricting loadings to be equal, showed a marginally decreased fit, supporting *full metric invariance* (ΔCFI = .002, ΔNCI = .006). Since, additionally restricting the thresholds, lead to decreased fit, full scalar invariance was not supported. To achieve *partial scalar invariance* according to the permissible changes in fit proposed by Cheung and Rensvold (2002), four item thresholds were allowed to vary across language groups, following an evaluation of the Lagrange Multiplier Test scores: The first thresholds from Item 10 (subscale Anger), Item 05 (Somatic Symptoms), and Item 13 (Sadness), and additionally the second threshold from Item 08 (Anger; Supplementary Table 3).
